# Predicting Risk Factors of Acute Kidney Injury in the First 7 Days after Admission: Analysis of a Group of Critically Ill Patients

**DOI:** 10.1155/2022/1407563

**Published:** 2022-12-21

**Authors:** Kexin Wen, Yongqing Huang, Qi Guo, Tao Wu, Juanzhang Liu, Yuping Zheng, Shuxian Zhou, Dengfeng Geng

**Affiliations:** Department of Cardiology, Sun Yat-sen Memorial Hospital, Sun Yat-sen University, Guangzhou, China

## Abstract

**Background:**

Acute kidney injury (AKI) is a common complication in critically ill patients. Some predictive models have been reported, but the conclusions are controversial. The aim of this study was the formation of nomograms to predict risk factors for AKI in critically ill patients within the first 7 days after admission to the intensive care unit (ICU).

**Methods:**

Data were extracted from the Medical Information Mart for Intensive Care- (MIMIC-) III database. The random forest method was used to fill in the missing values, and least absolute shrinkage and selection operator (Lasso) regression analysis was performed to screen for possible risk factors.

**Results:**

A total of 561 patients were enrolled. Complication with AKI is significantly associated with a longer length of stay (LOS). For all patients, the predictors contained in the prediction nomogram included hypertension, coronary artery disease (CAD), cardiopulmonary bypass (CPB), coronary artery bypass grafting (CABG), Simplified Acute Physiology Score II (SAPS II), central venous pressure (CVP) measured for the first time after admission, and maximum and minimum mean artery pressure (MAP). The model showed good discrimination (C − index = 0.818, 95% CI: 0.779-0.857). In the subgroup of patients with well-controlled blood glucose levels, the significant predictors included hypertension, CABG, CPB, SAPS II, and maximum and minimum MAP. Good discrimination was also present before (C − index = 0.785, 95% CI: 0.736–0.834) and after adjustment (adjusted C − index = 0.770).

**Conclusion:**

Hypertension, CAD, CPB, CABG, SAPS II, CVP measured for the first time after admission, and maximum and minimum MAP were independent risk factors for AKI in critically ill patients.

## 1. Introduction

Acute kidney injury (AKI) is a serious clinical complication featuring both attributed morbidity and mortality in the short and long term [1–3]. Currently, there are a series of diagnostic criteria for AKI, including the risk, injury, failure, loss, and end-stage renal disease (RIFLE) [4], AKI Network (AKIN) [5], and Kidney Disease: Improving Global Outcomes (KDIGO) [6] classifications. Among different populations, the diverse diagnostic criteria correspond to the different incidences of AKI, which vary from 1% to 70% [7–9]. Given its significantly adverse effect on the prognosis of critically ill patients, early detection of potential AKI is vital [10].

As one of the classic and traditional predictors of renal insufficiency, serum creatinine has been labeled as limited now because of its unstable level in critically ill patients. Previous research reported that some prediction models incorporate age, the baseline estimated glomerular filtration rate (eGFR), N-terminal brain natriuretic peptide precursor (NT-proBNP), and some of the drugs affect kidney function, such as metformin and angiotensin converting enzyme inhibitor (ACEI). [11] Some results are still controversial. Also, a series of novel biomarkers, including urinary hemojuvelin (uHJV), kidney injury molecule-1 (uKIM-1), and neutrophil gelatinase-associated lipocalin (uNGAL), were also studied to better predict the occurrence of AKI [12, 13]. However, these markers are rarely used in the clinic and lack sensitivity or specificity. Thus, it is vital to build models of efficiency that contain common clinical indicators to predict AKI. Then, nomograms are facilitated to visually exhibit the role of each risk factor.

In our earlier study, we had explored the potential heterogeneity of AKI and evaluated the prognostic differences among AKI subphenotypes in critically ill patients with cardiovascular diseases [14]. In this study, we further built several nomograms of prediction models for different populations of AKI patients. The data were obtained from the Medical Information Mart for Intensive Care- (MIMIC-) III database, which is composed of a large amount of clinical and test data collected from the ICU [15].

## 2. Methods

### 2.1. Data Source

The data in this study were extracted from the MIMIC-III (Medical Information Mart for Intensive Care) database established by Beth Israel Deaconess Medical Center in Boston, Massachusetts, USA [15]. This is a large, single-center database that is comprised of information relating to the patients admitted to critical care units and includes patient demographics, vital signs, medications, laboratory measurements, fluid balance, procedure and diagnostic codes, imaging reports, hospital length of stay, and death events. The use of the MIMIC-III database in this study was approved by the review committee of Massachusetts Institute of Technology and Beth Israel Deaconess Medical Center.

### 2.2. Study Population

Patients older than 18 years old with a length of ICU stay longer than one day were included. For the patients who were recorded with multiple admissions, only the first ICU admission was extracted. Ultimately, 561 patients in the critical care units were enrolled for the following analysis.

### 2.3. Covariates and Outcomes

The baseline characteristics were extracted within the initial 24 hours after critical care unit admission. The covariates in this study included age, sex, body mass index (BMI), heart rate (HR), respiratory rate, oxygen saturation (SpO2), temperature, glucose, systolic blood pressure (SBP), diastolic blood pressure (DBP), 24-hour urine output, use of ventilation, stage of acute kidney injury at 48 hours and 7 days after admission, administration of vasopressors, sedatives, and furosemide.

The comorbidities included coronary artery disease (CAD), atrial fibrillation (AF), congestive heart failure (CHF), hypertension, stroke, sepsis, diabetes mellitus (DM), and chronic obstructive pulmonary disease (COPD), which were all recorded as International Classification of Diseases, Ninth Revision (ICD-9) codes. The procedures included cardiopulmonary bypass, coronary artery bypass grafting (CABG), and left heart catheterization.

The laboratory test measurements included the white blood cell (WBC) count and levels of hemoglobin, platelets, sodium, potassium, blood urea nitrogen (BUN), and creatinine. The severity at admission was measured by the Sequential Organ Failure Assessment (SOFA) score, Simplified Acute Physiology Score (SAPS), Simplified Acute Physiology Score II (SAPS II), Elixhauser comorbidity score, and length of ICU stay. The outcome of the current study was AKI.

### 2.4. Statistical Analyses

Continuous variables are presented as the SEM ± SD or median (interquartile range), and categorical variables are presented as numbers (percentages). The random forest method was used to fill in the missing values, and Student's *t*-test was used to compare significant differences in the data before and after filling. The chi-square test and the Kruskal–Wallis test were used for comparisons among the groups. After univariate binary logistic regression analysis, the least absolute shrinkage and selection operator (LASSO) method, which is suitable for the reduction in high-dimensional data [16, 17], was applied to select the optimal predictive features in the risk factors of critically ill patients. Eighty percent of the sample data were randomly selected as the training set and the remaining 20% as the fitting model of the test set. The odds ratio (OR) and 95% confidence interval (CI) values were determined by multivariate logistic regression to establish the final model. A two-tailed *P*value < 0.05 was considered statistically significant. Statistical analyses were carried out by using SPSS (version 25.0, IBM, New York, USA) and the R tool (version 4.0.2, R Foundation for Statistical Computing, Vienna, Austria).

## 3. Results

### 3.1. In All Statistics

#### 3.1.1. Patient Characteristics

A total of 561 patients, consisting of 440 (78.43%) patients who were diagnosed with AKI in the first 7 days after admission to the ICU, were finally included in this study. There was no significant difference between the variables before and after filling. Their baseline information, including demographic, disease, and treatment features, in the two groups is shown in Table 1.

#### 3.1.2. Feature Selection and Drawing of ROC Curve

**Figure 1:**
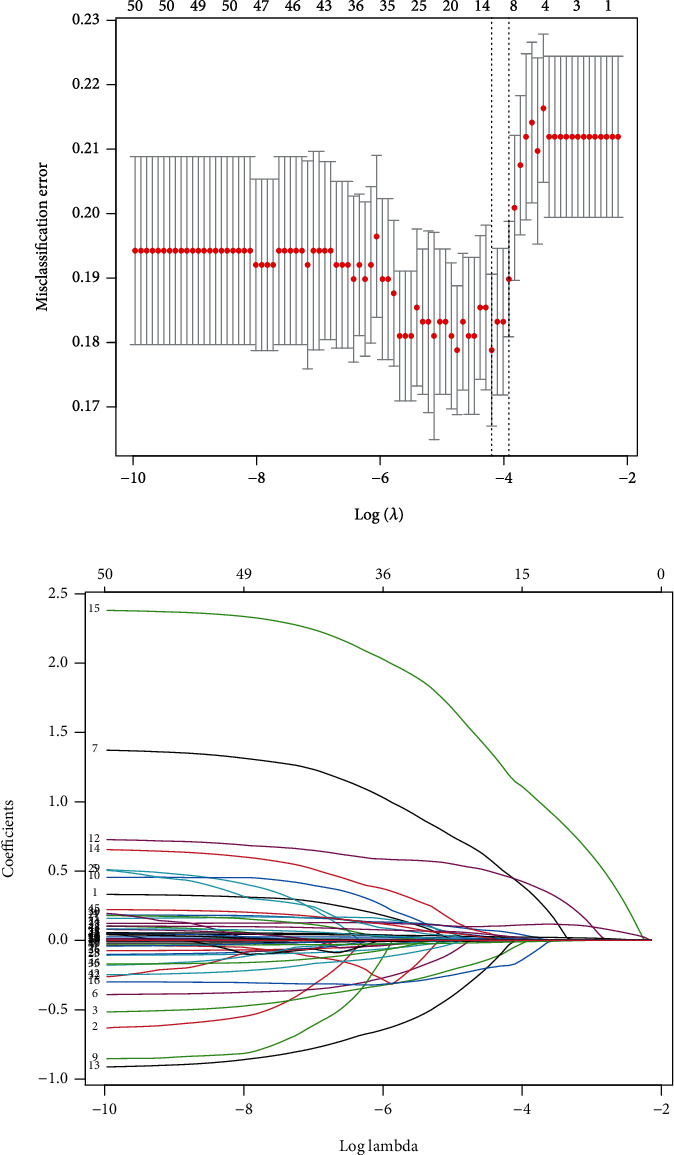
Selection of the demographic and clinical features by using the LASSO binary logistic regression model. Notes: (a) Optimal parameter (lambda) selection in the LASSO model using fivefold cross-validation via minimum criteria. The partial likelihood deviance (binomial deviance) curve was plotted versus log(lambda). Dotted vertical lines were drawn at the optimal values by using the minimum criteria and the 1 SE of the minimum criteria (the 1-SE criteria). (b) LASSO coefficient profiles of the 51 features. A coefficient profile plot was produced against the log(lambda) sequence. A vertical line was drawn at the value selected using fivefold cross-validation, where optimal lambda resulted in five features with nonzero coefficients. Abbreviations: LASSO: least absolute shrinkage and selection operator; SE: standard error.

**Figure 2:**
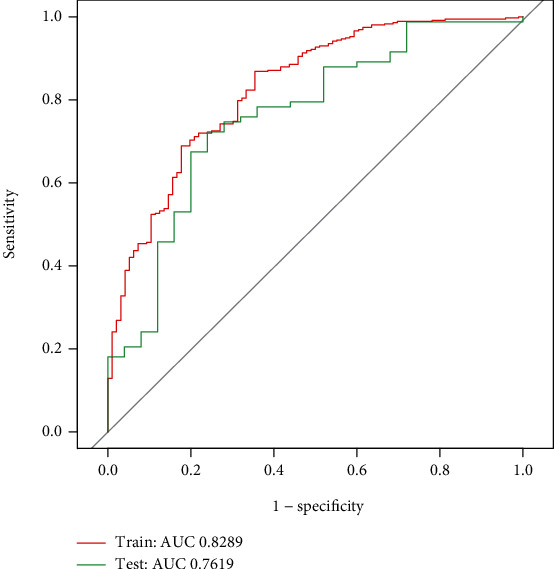
Developed ROC curve of the risk factors in the training set and test set. Abbreviations: ROC: receiver operator characteristic; AUC: area under the curve.

After the univariate binary logistic regression analysis, 51 features with a *P*value < 0.1 were put into the LASSO regression model for further screening, as 80% of the data were randomly selected as the training set, and the remaining 20% were selected as the test set (Figures 1(a) and 1(b)). The ROC curves and AUC presented good predictive value of the model (Figure 2). The features included hypertension, CAD, cardiopulmonary bypass, CABG, SAPS II, CVP measured for the first time after admission, and maximum and minimum MAP during the ICU stay (Table 2).

#### 3.1.3. Development of the Prediction Model

**Figure 3:**
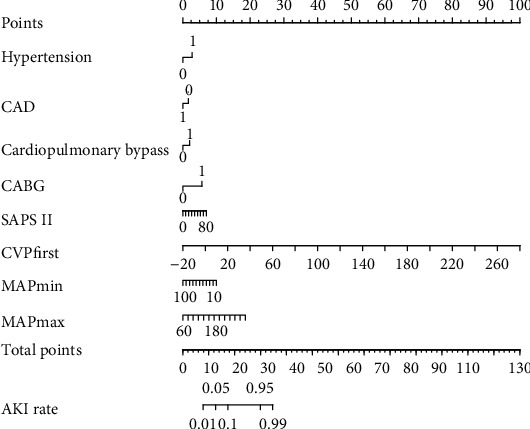
The developed medication nonadherence nomogram. Abbreviations: CAD: coronary artery disease; CABG: coronary artery bypass surgery; SAPS II: Simplified Acute Physiology Score II; CVP: central venous pressure; MAP: mean arterial pressure; AKI: acute kidney injury.

The results of multivariate logistic regression analysis are presented in Table 2. The model that incorporated the above independent predictors was developed and presented as the nomogram (Figure 3).

#### 3.1.4. Apparent Performance of the Nonadherence Risk Nomogram in the Cohort

The C-index for the prediction nomogram was 0.818 (95% CI: 0.779-0.857) for the cohort and was confirmed to be 0.802 through bootstrapping validation, suggesting the model's good discrimination.

### 3.2. In the HbA1c < 6.5% Subgroup

#### 3.2.1. Patient Characteristics

The HbA1c < 6.5% subgroup contained 324 (73.63%) patients with AKI in the first 7 days after admission to the ICU. Their baseline information, including demographics, diseases, and treatment features, in the two groups is shown in Table 3.

#### 3.2.2. Feature Selection and Drawing of the ROC Curve

**Figure 4:**
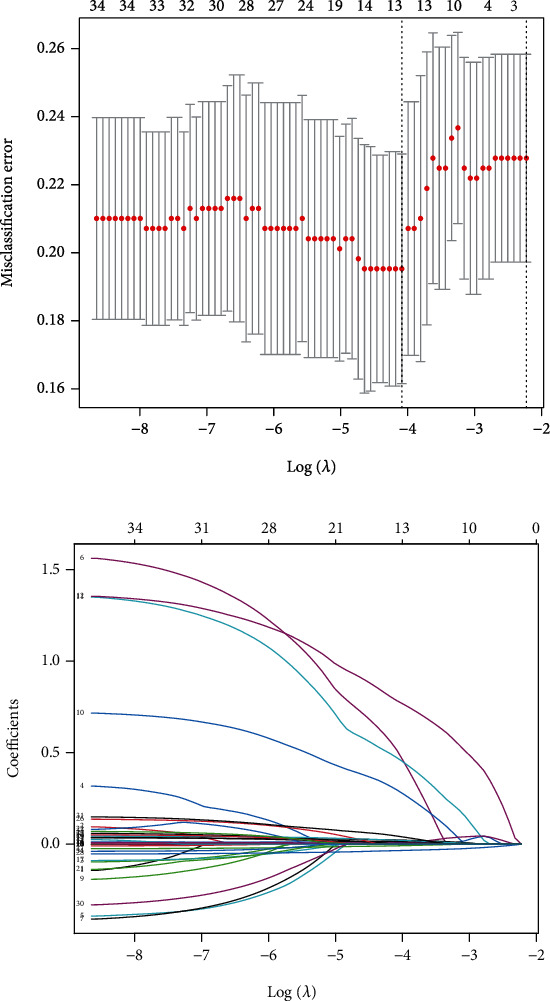
Selection of the demographic and clinical features in the HbA1c < 6.5% subgroup by using the LASSO binary logistic regression model. Notes: (a) Optimal parameter (lambda) selection in the LASSO model using fivefold cross-validation via minimum criteria. The partial likelihood deviance (binomial deviance) curve was plotted versus log(lambda). Dotted vertical lines were drawn at the optimal values by using the minimum criteria and the 1 SE of the minimum criteria (the 1-SE criteria). (b) LASSO coefficient profiles of the 34 features. A coefficient profile plot was produced against the log(lambda) sequence. A vertical line was drawn at the value selected using fivefold cross-validation, where the optimal lambda resulted in five features with nonzero coefficients. Abbreviations: LASSO: least absolute shrinkage and selection operator; SE: standard error.

**Figure 5:**
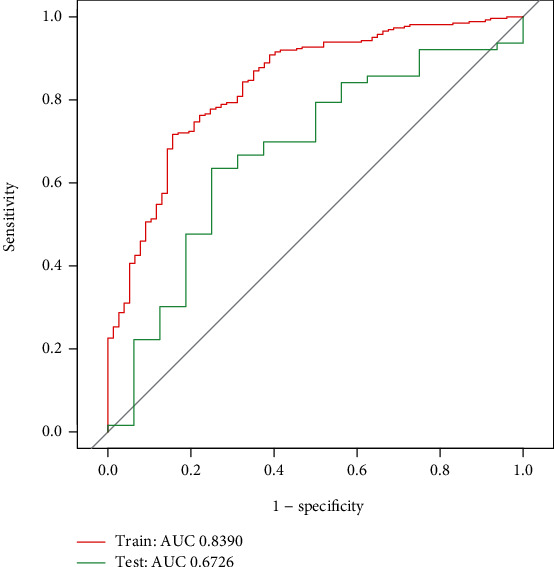
Developed ROC curve of risk factors in the training set and test set in the HbA1c < 6.5% subgroup. Abbreviations: ROC: receiver operator characteristic; AUC: area under the curve.

Among the demographic, disease, and laboratory examination indexes, 34 features were put into the LASSO regression model for further screening, as 80% of the data were randomly selected as the training set and the remaining 20% as the test set (Figures 4(a) and 4(b)). The ROC curves and AUC presented good predictive value of the model (Figure 5). The features included hypertension, CABG, cardiopulmonary bypass, SAPS II, and maximum and minimum MAP during the ICU stay (Table 3).

#### 3.2.3. Development of the Prediction Model

**Figure 6:**
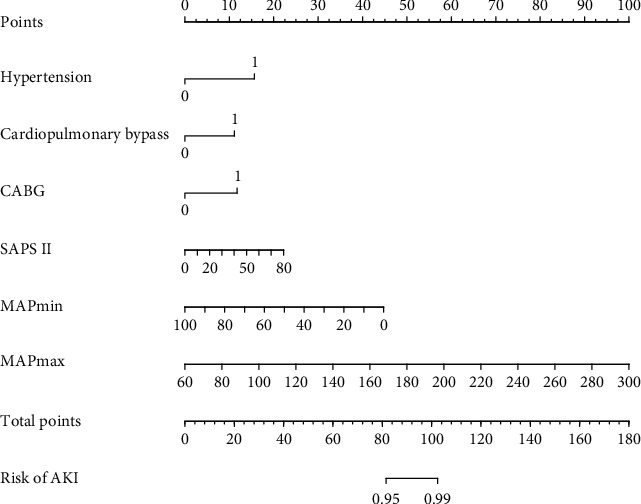
The developed medication nonadherence nomogram in the HbA1c < 6.5% subgroup. Abbreviations: CABG: coronary artery bypass surgery; SAPS II: Simplified Acute Physiology Score II; MAP: mean arterial pressure; AKI: acute kidney injury.

The results of the multivariate logistic regression analysis are presented in Table 4. The model that incorporated the above independent predictors was developed and presented as the nomogram (Figure 6).

#### 3.2.4. Apparent Performance of the Nonadherence Risk Nomogram in the Cohort

The C-index for the prediction nomogram was 0.785 (95% CI: 0.736–0.834) for the cohort and was confirmed to be 0.770 through bootstrapping validation, suggesting the model's good discrimination.

## 4. Discussion

In this retrospective observational study, we reported an overall AKI incidence of 78.43% among a population of critically ill patients and an incidence of 73.63% in the subgroup of HbA1c < 6.5. Additionally, we built two nomograms for the critically ill patients enrolled and the subgroup of patients with HbA1c < 6.5. The predictive value of the models performed well in both the training set and test set. Even after adjustment, good discrimination still exists. The combination of clinical markers could better anticipate the development of AKI, helping doctors to be vigilant and eventually achieve further prevention of AKI.

AKI is a major complication with quite a high incidence in the ICU and is associated with increased treatment expenses and longer hospital stays [18]. Our study confirmed the high incidence of AKI in critically ill patients, which has been reported in other studies [19, 20]. The patients complicated with AKI had a higher 28-day mortality and increased LOS, suggesting the necessity to take strategies to reduce the morbidity of AKI. Although a series of studies have identified risk factors for AKI, there is still room for the development of risk prediction tools for AKI in critically ill patients.

There have been a series of predictive models found in patients with contrast-induced AKI [21], underwent surgery [22], after liver transplantation [23], and with sepsis [24]. Recently, the standardized diagnostic and staging criteria for AKI have contributed to an improved understanding of the incidence and course of AKI in critically ill patients. However, there is still variation in its timely recognition, management, and outcomes [1, 20]. There are novel biomarkers showing predictive value for AKI, including cystatin C, neutrophil gelatinase-associated lipocalin, interleukin-18, protein C, insulin-like growth factor-binding protein 7, tissue inhibitor of metalloproteinases-2, and kidney injury molecule-1. However, the sensitivity and specificity of these indicators are still high, and they are not routinely measured in clinical diagnosis and treatment work. The relatively high costs of the assays are another disadvantage.

To predict the occurrence of AKI more effectively and reduce its harm to patients, a combination of epidemiological, clinical, biological, and hereditary factors along with a series of biomarkers is required to be included in the ideal predictive models [25]. Medical Information Mart for Intensive Care (MIMIC), as one of public database of high quality, is widely used in evaluating clinical risks and building disease-prediction models owing to advantages of being highly valuable for data mining [26, 27]. Moreover, nomograms are widely used as prognostic devices in medicine. Applying multivariate logistic regression to build nomograms is accurate enough to help to better achieve user-friendly digital interfaces and more easily understood prognoses to make better clinical decisions [28].

In this study, data of 561 patients from MIMIC were used to build the models. It was found that hypertension, CAD, cardiopulmonary bypass, CABG, SAPS II, CVP measured for the first time after admission, and the maximum and minimum MAP during the ICU stay were significantly associated with an increased risk of AKI for critically ill patients in the first 7 days after admission. We founded two models of AKI with satisfactory AUCs of 0.762 and 0.672 and adjusted C-indexes of 0.802 and 0.770. After incorporated into nomograms, these clinical risk factors facilitated the prediction of AKI and possessed satisfying predictive value. Gujadhur et al. [29] reported a model from data of more than 2000 patients from intensive care unit (ICU), to predict development of AKI. The multiregression model included serum bicarbonate on admission (OR = 0.821; 95% CI: 0.796-0.846; *P* < 0.0001), APACHE III (OR = 1.011; 95% CI: 1.007-1.015; *P* < 0.0001), age (OR = 1.016; 95% CI 1.008-1.024; *P* < 0.0001), and presence of sepsis at ICU admission (OR = 2.819; 95% CI: 2.122-23.744; *P* = 0.004), with an AUC of 0.8 (95% CI: 0.78-0.83). In our model, we also showed the association between a classic score which commonly used to assess physiological status of critically ill patients, SAPS II, and AKI. Another model was developed, which contained heart failure, chronic kidney disease, emergency surgery, sepsis, and total bilirubin, to predict occurrence of critically ill patients, and the AUC (0.81), sensitivity (69.8%), and specificity (83.4%) were satisfactory [30]. Compared with Gujadhur A's model, this model contained more disease variables, which is also a feature of our model.

Published in 1993, the Simplified Acute Physiology Score (SAPS) II was developed and validated in a European and North American cohort and includes 17 variables collected during the first 24 hours of ICU stay [31]. The sum of the score represents the in-hospital mortality risk, and its predictive performance has been evaluated in multiple studies [32, 33]. Xu et al. also used the data from postcardiac surgery patients from the MIMIC-III database and reported better discriminative performances of both the 90-day mortality and 1-year mortality of the SAPS II scoring system than the SOFA scoring system [34]. SAPS II has been widely reported as an independent risk factor for AKI in different populations and different stages of AKI [35–37]. In our study, a similar positive result was concluded, suggesting the predictive value of SAPS II for the outcomes for critical patients.

AKI occurs in 2% to 30% of patients undergoing cardiac surgery [38]. It is largely assumed that the pathologic lesion of AKI after cardiac surgery is acute tubular necrosis [39]. The injured tubular epithelial cells slough, resulting in intratubular obstruction and hypertension. After the appearance of alterations in vasoreactivity, prerenal azotemia occurs, and cellular ATP depletion and oxidative injury eventually contribute to AKI. CPB is an intraoperative event associated with significant hemodynamic changes. Ischemic-reperfusion injury is common following cardiopulmonary bypass (CPB) and causes AKI [40]. In a meta-analysis that enrolled 46 studies comprising 242,388 participants [41], a significant association between CPB under different diagnostic criteria for AKI was reported. Minute oxygen consumption (VO2) and perfusion pressure during CPB are the two major determinants affecting the local hemodynamics of the kidney. The steady, nonpulsatile nature of CPB negates the elastance, inertial, and reflective components of the arterial resistance during normal pulsatile flow, making the regulation of local perfusion pressure more important. However, the optimum parameters of CPB flow and pressure goals are not known. Moderately high levels were reported to be associated with a reduced incidence of cardiac and neurologic complications, but the renal function was not assessed simultaneously [42].

It is worth noting that the relationship between CAD and AKI was anomalous. Yayan [43] compared occurrence of AKI after PCI of patients with and without CAD. Results showed that the occurrence of AKI was not significantly related to the presence of coronary heart disease (*P* = 0.95, chi-square test). In our study, the baseline presence of CAD and AKI is not associated (*P* = 0.050, chi-square test), neither. And after Lasso regression and multiple regression analysis, CAD was positively related to AKI. Combined with the results of this study, we speculated the difference of definitions and diagnostic criteria of AKI led to this abnormal result. However, there being no research elaborated the nonsignificant even positive relationship between CAD and AKI, future studies featured as more patients and prospective data collection with diagnostic criteria of AKI more applicable to Chinese are needed to solve this problem.

We believe our work provides clinicians with a new tool to identify patients with a high risk of AKI and the requirements of preventive strategies. The risk scores based on the parameters that are available to clinicians are higher, as they are faster and easier to obtain. Moreover, cheaper and more valid biomarkers, whose level is less affected by other factors, still need to be explored to evaluate changes in renal function. More sophisticated and effective models to predict AKI are needed for the prevention and intervention of adverse outcomes. There were several limitations that should be mentioned in our current study. First, the cohort could not represent all critically ill patients as those who without access to treatment were not included. Second, all comorbidities were recorded by ICD-9 codes, which might satisfy the latest diagnostic criteria for some diseases. Third, in all critically ill patients included in our study, CAD is a protective factor. As being analyzed, it is because of the inevitable deficiencies of the retrospective design, such as failure to assess patients' status in a timely manner and limitations on the number of cases. Therefore, future studies featured as more patients and prospective data collection are needed to help enhance the credibility of our results.

## 5. Conclusion

This study applied a novel nomogram with relatively good accuracy to assess the risk of AKI in critically ill patients. Eventually, hypertension, CAD, cardiopulmonary bypass, CABG, SAPS II, CVP measured for the first time after admission, and maximum and minimum MAP during the ICU stay were independent risk factors for AKI for critically ill patients within the first 7 days of admission. Further study is needed to reveal the potential mechanisms.

## Figures and Tables

**Table 1:** Differences between the demographic and clinical characteristics of the two groups.

Demographic characteristics	No-AKI (*n* = 121)	AKI (*n* = 440)	*P* value
Height (cm)	167.94 ± 10.33	170.26 ± 10.06	0.026
BMI (m/kg^2^)	28.56 ± 6.63	29.55 ± 6.27	0.129
APS III	33.91 ± 13.29	41.53 ± 18.04	<0.001
First wardid	12 (8)	14 (3)	0.320
Last wardid	14 (8)	14 (3)	0.687
LOS	1.64 (1.49)	2.39 (3.41)	<0.001
Age	62.41 ± 15.90	66.46 ± 13.37	0.011
Weight	79.96 ± 22.74	85.41 ± 20.01	0.010
SAPS	16.73 ± 4.61	19.46 ± 4.64	<0.001
SOFA	3.08 ± 2.38	4.80 ± 2.73	<0.001
SAPS II	28.67 ± 11.19	36.17 ± 12.56	<0.001
Elix score	2.45 ± 5.44	3.40 ± 5.68	0.098
Elixhauser vanwalraven	2.31 ± 4.98	3.59 ± 6.11	0.019
Elixhauser_sid29	2.74 ± 6.24	4.86 ± 8.13	0.002
Elixhauser_sid30	4.17 ± 8.59	6.82 ± 10.45	0.005
Mingcs	15 (1)	15 (0)	0.163
GCS_motor_	6 (1)	6 (5)	0.010
GCS_verbal_	5 (4.5)	1.85 (5)	0.001
GCS_eyes_	4 (1)	3 (3)	0.001
GCS_total_	14 (6)	10 (12)	0.001
Glucose_min_ (mmol)	5.75 ± 1.83	5.39 ± 1.72	0.050
Glucose_max_ (mmol)	9.67 ± 4.08	10.55 ± 3.80	0.027
Glucose_mean_ (mmol)	7.50 ± 2.29	7.63 ± 2.02	0.540
Glucose_range_ (mmol)	3.93 ± 3.95	5.16 ± 3.84	0.002
HbA1c (%)	6.33 ± 1.63	6.58 ± 1.93	0.193
HR_first_ (bpm)	84.27 ± 17.42	85.65 ± 16.22	0.416
HR_min_ (bpm)	64.53 ± 12.07	65.20 ± 12.64	0.602
HR_max_ (bpm)	103.78 ± 20.08	111.94 ± 22.16	<0.001
CVP_first_ (cmH_2_O)	10.50 ± 4.33	12.64 ± 13.28	0.082
CVP_min_ (cmH_2_O)	4.10 ± 3.49	5.45 ± 13.21	0.266
MAP_first_ (mmHg)	84.21 ± 15.66	79.64 ± 16.06	0.006
MAP_min_ (mmHg)	56.55 ± 11.78	50.20 ± 12.82	<0.001
MAP_max_ (mmHg)	104.33 ± 16.59	113.60 ± 27.17	<0.001
Temperature_first_ (°C)	36.44 ± 0.90	36.22 ± 0.83	0.013
Temperature_min_ (°C)	35.92 ± 0.60	35.66 ± 0.96	0.006
Temperature_max_ (°C)	37.82 ± 1.83	39.66 ± 23.67	0.392
Hemoglobin_first_ (g/L)	10.94 ± 2.09	10.52 ± 2.19	0.064
Hemoglobin_min_ (g/L)	10.47 ± 2.12	9.55 ± 2.07	<0.001
Hemoglobin_max_ (g/L)	11.62 ± 1.80	11.50 ± 1.68	0.475
WBC_first_ (×10^9/L)	11.73 ± 4.82	12.81 ± 5.84	0.063
WBC_min_ (×10^9/L)	10.45 ± 4.04	11.52 ± 5.15	0.036
WBC_max_ (×10^9/L)	12.40 ± 4.92	14.13 ± 6.16	0.005
PLT_first_ (×10^9/L)	212.58 ± 91.50	190.16 ± 90.12	0.016
PLT_min_ (×10^9/L)	199.92 ± 91.25	175.74 ± 85.32	0.007
PLT_max_ (×10^9/L)	221.23 ± 91.67	210.56 ± 86.29	0.235
Angus sepsis, *n* (%)	12 (10)	83 (19)	0.029
Sedative, *n* (%)	51 (42)	307 (70)	<0.001
Gender, *n* (%)	69 (57)	299 (68)	0.033
SIRS			0.005
0	3 (2)	3 (1)	
1	21 (17)	32 (7)	
2	31 (26)	107 (24)	
3	37 (31)	165 (38)	
4	29 (24)	133 (30)	
Ventilation, *n* (%)	54 (45)	327 (74)	<0.001
Vasoactive drugs, *n* (%)	43 (36)	264 (60)	<0.001
Congestive heart failure, *n* (%)	3 (2)	25 (6)	0.231
Cardiac arrhythmias, *n* (%)	5 (4)	29 (7)	0.430
Valvular disease, *n* (%)	1 (1)	10 (2)	0.471
Pulmonary circulation, *n* (%)	2 (2)	4 (1)	0.614
Peripheral vascular, *n* (%)	14 (12)	56 (13)	0.853
Hypertension, *n* (%)	6 (5)	57 (13)	0.021
Paralysis, *n* (%)	4 (3)	8 (2)	0.299
Other neurological, *n* (%)	4 (3)	20 (5)	0.731
Chronic pulmonary, *n* (%)	14 (12)	71 (16)	0.272
Uncomplicated diabetes, *n* (%)	25 (21)	128 (29)	0.084
Complicated diabetes, *n* (%)	3 (2)	21 (5)	0.395
All diabetes, *n* (%)	28 (23)	149 (34)	0.033
CHF, *n* (%)	28 (23)	141 (32)	0.075
Atrial fibrillation, *n* (%)	25 (21)	154 (35)	0.004
Renal disease, *n* (%)	9 (7)	47 (11)	0.377
COPD, *n* (%)	7 (6)	43 (10)	0.237
CAD, *n* (%)	64 (53)	276 (63)	0.063
Stroke, *n* (%)	10 (8)	26 (6)	0.467
Malignancy, *n* (%)	10 (8)	39 (9)	0.980
Cardiopulmonary bypass, *n* (%)	36 (30)	259 (59)	<0.001
CABG, *n* (%)	17 (14)	207 (47)	<0.001
Left heart catheterization, *n* (%)	54 (45)	159 (36)	0.110

Abbreviations: BMI: body mass index; APS III: autoimmune polyglandular syndrome type III; LOS: length of stay; SAPS: Simplified Acute Physiology Score; SOFA: Sequential Organ Failure Assessment; SAPS II: Simplified Acute Physiology Score II; GCS: Glasgow coma scale; HbA1c: hemoglobin A1c; HR: heart rate; CVP: central venous pressure; MAP: mean arterial pressure; WBC: white blood cell; PLT: platelet; SIRS: systemic inflammatory response syndrome; CHF: chronic heart failure; COPD: chronic obstructive pulmonary disease; CAD: coronary artery disease; CABG: coronary artery bypass grafting.

**Table 2:** Prediction factors for AKI in critically ill patients.

Variable	*β*	OR	95% CI	*P* value
Hypertension	1.035	2.816	1.071-7.403	0.036
CAD	-0.72	0.487	0.271-0.874	0.016
Cardiopulmonary bypass	0.637	1.891	1.002-3.568	0.049
CABG	1.923	6.843	2.902-16.14	<0.001
SAPS II	0.037	1.038	1.013-1.064	0.003
CVP_first_	0.1	1.105	1.042-1.171	0.001
MAP_min_	-0.037	0.963	0.941-0.986	0.002
MAP_max_	0.028	1.029	1.012-1.046	0.001

Note: *β* is the regression coefficient. Abbreviations: AKI: acute kidney injury; CAD: coronary artery disease; CABG: coronary artery bypass surgery; SAPS II: Simplified Acute Physiology Score II; CVP: central venous pressure; MAP: mean arterial pressure; OR: odds ratio; CI: confidence interval.

**Table 3:** Differences between the demographic and clinical characteristics of the two groups in the HbA1c < 6.5% subgroup.

Demographic variables	No-AKI (*n* = 93)	AKI (*n* = 324)	*P* value
Height (cm)	168.00 ± 10.27	171.25 ± 9.54	0.005
BMI (m/kg^2^)	28.38 ± 7.15	29.45 ± 6.28	0.164
APS III	35.18 ± 13.66	42.18 ± 18.69	<0.001
First wardid	12 (8)	14 (3)	0.393
Last wardid	14 (8)	14 (3)	0.968
Age	63.11 ± 15.35	65.62 ± 13.45	0.126
Weight	79.52 ± 24.46	86.02 ± 20.09	0.009
SAPS	17.30 ± 4.68	19.37 ± 4.68	<0.001
SOFA	3.32 ± 2.50	4.88 ± 2.86	<0.001
SAPS II	29.77 ± 11.78	36.31 ± 13.05	<0.001
Elix score	2.77 ± 5.74	3.40 ± 5.87	0.359
Elixhauser vanwalraven	2.60 ± 5.11	3.56 ± 6.15	0.169
Elixhauser_sid29	3.16 ± 6.39	4.70 ± 7.97	0.055
Elixhauser_sid30	4.78 ± 8.94	6.74 ± 10.41	0.076
Mingcs	15 (1)	15 (0)	0.072
GCS_motor_	6 (2)	6 (5)	0.136
GCS_verbal_	5 (5)	1 (5)	0.020
GCS_eyes_	4 (1)	3 (3)	0.043
GCS_total_	14 (8)	10 (12)	0.057
Glucose_min_ (mmol)	5.64 ± 1.92	5.43 ± 1.75	0.329
Glucose_max_ (mmol)	9.97 ± 4.39	10.39 ± 3.74	0.356
Glucose_mean_ (mmol)	7.56 ± 2.48	7.60 ± 2.07	0.887
Glucose_range_ (mmol)	4.33 ± 4.21	4.96 ± 3.76	0.167
HbA1c (%)	5.71 ± 0.35	5.75 ± 0.36	0.369
HR_first_ (bpm)	84.34 ± 17.17	86.05 ± 16.86	0.392
HR_min_ (bpm)	64.88 ± 12.75	65.14 ± 12.85	0.866
HR_max_ (bpm)	104.53 ± 19.69	112.82 ± 22.76	0.002
CVP_first_ (cmH_2_O)	10.71 ± 4.31	12.83 ± 15.19	0.184
CVP_min_ (cmH_2_O)	4.05 ± 3.52	5.77 ± 15.25	0.282
MAP_first_ (mmHg)	84.19 ± 16.51	79.95 ± 16.12	0.027
MAP_min_ (mmHg)	56.19 ± 11.43	49.66 ± 13.00	<0.001
MAP_max_ (mmHg)	104.29 ± 16.58	113.83 ± 25.99	0.001
Temperature_first_ (°C)	36.42 ± 0.89	36.27 ± 0.84	0.125
Temperature_min_ (°C)	35.89 ± 0.61	35.67 ± 1.04	0.057
Temperature_max_ (°C)	37.84 ± 1.73	38.50 ± 2.93	0.041
Hemoglobin_first_ (g/L)	10.86 ± 2.04	10.58 ± 2.23	0.289
Hemoglobin_min_ (g/L)	10.33 ± 2.08	9.57 ± 2.11	0.002
Hemoglobin_max_ (g/L)	11.61 ± 1.82	11.52 ± 1.73	0.686
WBC_first_ (×10^9/L)	11.87 ± 5.13	12.88 ± 5.77	0.129
WBC_min_ (×10^9/L)	10.52 ± 4.23	11.52 ± 4.85	0.073
WBC_max_ (×10^9/L)	12.57 ± 5.24	14.11 ± 5.94	0.025
PLT_first_ (×10^9/L)	207.40 ± 86.37	192.05 ± 89.12	0.141
PLT_min_ (×10^9/L)	193.76 ± 86.57	178.97 ± 87.06	0.149
PLT_max_ (×10^9/L)	216.35 ± 86.40	211.41 ± 86.27	0.627
Angus sepsis, *n* (%)	12 (13)	61 (19)	0.242
Sedative, *n* (%)	44 (47)	226 (70)	<0.001
Gender, *n* (%)	55 (59)	231 (71)	0.036
SIRS, *n* (%)			0.016
Ventilation, *n* (%)	47 (51)	244 (75)	<0.001
Vasoactive drugs, *n* (%)	37 (40)	196 (60)	<0.001
Congestive heart failure, *n* (%)	3 (3)	16 (5)	0.586
Cardiac arrhythmias, *n* (%)	5 (5)	23 (7)	0.726
Valvular disease, *n* (%)	1 (1)	8 (2)	0.691
Pulmonary circulation, *n* (%)	2 (2)	3 (1)	0.310
Peripheral vascular, *n* (%)	12 (13)	40 (12)	1.000
Hypertension, *n* (%)	5 (5)	43 (13)	0.055
Paralysis, *n* (%)	4 (4)	6 (2)	0.240
Other neurological, *n* (%)	3 (3)	16 (5)	0.586
Chronic pulmonary, *n* (%)	11 (12)	50 (15)	0.484
Diabetes uncomplicated, *n* (%)	20 (22)	95 (29)	0.175
Diabetes complicated, *n* (%)	2 (2)	14 (4)	0.541
Diabetes all, *n* (%)	22 (24)	109 (34)	0.089
Furosemide, *n* (%)	11 (12)	37 (11)	1.000
CHF, *n* (%)	22 (24)	108 (33)	0.099
Atrial fibrillation, *n* (%)	21 (23)	120 (37)	0.013
Renal disease, *n* (%)	7 (8)	35 (11)	0.466
Liver disease, *n* (%)	1 (1)	8 (2)	0.691
COPD, *n* (%)	5 (5)	31 (10)	0.289
CAD, *n* (%)	50 (54)	199 (61)	0.227
Stroke, *n* (%)	8 (9)	24 (7)	0.872
Malignancy, *n* (%)	8 (9)	32 (10)	0.866
Cardiopulmonary bypass, *n* (%)	31 (33)	193 (60)	<0.001
CABG, *n* (%)	17 (18)	151 (47)	<0.001
Left heart catheterization, *n* (%)	36 (39)	121 (37)	0.906

Abbreviations: BMI: body mass index; APS III: autoimmune polyglandular syndrome type III; LOS: length of stay; SAPS: Simplified Acute Physiology Score; SOFA: Sequential Organ Failure Assessment; SAPS II: Simplified Acute Physiology Score II; GCS: Glasgow coma scale; HbA1c: Hemoglobin A1c; HR: heart rate; CVP: central venous pressure; MAP: mean arterial pressure; WBC: white blood cell; PLT: platelet; SIRS: systemic inflammatory response syndrome; CHF: chronic heart failure; COPD: chronic obstructive pulmonary disease; CAD: coronary artery disease; CABG: coronary artery bypass grafting.

**Table 4:** Prediction factors for AKI in the critically ill patients in the HbA1c < 6.5% subgroup.

Variable	*β*	OR	95% CI	*P* value
Hypertension	1.353	3.868	1.314-11.383	0.014
Cardiopulmonary bypass	0.794	2.213	1.126-4.347	0.021
CABG	0.897	2.452	1.159-5.189	0.019
SAPS II	0.028	1.029	1.003-1.054	0.026
MAP_min_	-0.044	0.957	0.933-0.983	0.001
MAP_max_	0.033	1.034	1.015-1.053	<0.001

Note: *β* is the regression coefficient. Abbreviations: CABG: coronary artery bypass surgery; SAPS II: Simplified Acute Physiology Score II; MAP: mean arterial pressure; OR: odds ratio; CI: confidence interval.

## Data Availability

The data used to support the findings of this study are included within the article.
